# Identification and *in silico* analysis of the origin recognition complex in the human fungal pathogen *Candida albicans*

**DOI:** 10.17912/micropub.biology.000465

**Published:** 2021-09-21

**Authors:** Sreedevi Padmanabhan, Kaustuv Sanyal, Dharanidhar Dubey

**Affiliations:** 1 Molecular Biology Laboratory, Veer Bahadur Singh Purvanchal University, Jaunpur- 222003, Uttar Pradesh, India.; 2 Molecular Mycology Laboratory, Molecular Biology and Genetics Unit, JNCASR, Bangalore - 560064, India.

## Abstract

DNA replication in eukaryotes is initiated by the orchestrated assembly and association of initiator proteins (heterohexameric Origin Recognition Complex, ORC) on the replication origins. These functionally conserved proteins play significant roles in diverse cellular processes besides their central role in ignition of DNA replication at origins. *Candida albicans*, a major human fungal pathogen, is a diploid budding yeast that belongs to Ascomycota. However, *C. albicans* is significantly diverged from a well-studied model organism *Saccharomyces cerevisiae*, another ascomycete. The components of the DNA replication machinery in *C. albicans* remain largely uncharacterized. Identification of factors required for DNA replication is essential for understanding the evolution of the DNA replication machinery. We identified the putative ORC homologs in *C. albicans* and determined their relatedness with those of other eukaryotes including several yeast species. Our extensive *in silico* studies demonstrate that the domain architecture of CaORC proteins share similarities with the ORC proteins of *S. cerevisiae*. We dissect the domain organization of ORC (trans-acting factors) subunits that seem to associate with DNA replication origins in *C. albicans. *We present a model of the 3D structure of CaORC4 to gain further insights of this protein’s function.

**Figure 1. Evolutionary relationship of CaORC proteins with other species, comparative domain architecture of CaORC and ScORC proteins and ORC phylogeny in CTG clade; 3D model of CaORC4:**
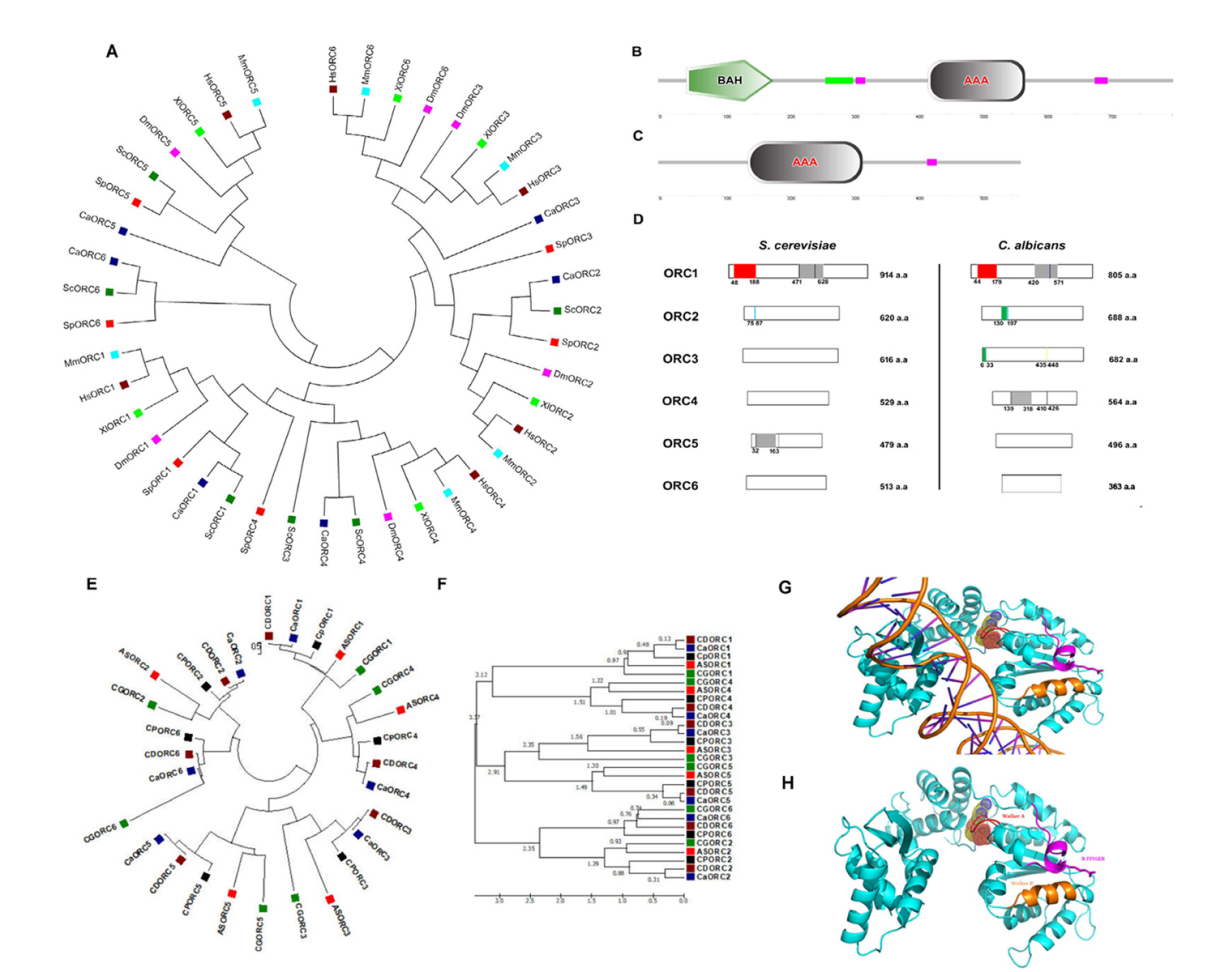
(A) Phylogram of ORC proteins. The tree is drawn to scale, with branch lengths in the same units as those of the evolutionary distances used to infer the phylogenetic tree. The evolutionary distances were computed using the Poisson correction method (Zuckerkandl and Pauling, 1965) and are in the units of the number of amino acid substitutions per site. All positions containing gaps and missing data were eliminated from the dataset (Complete deletion option). The optimal tree with the sum of branch length = 29.06 is shown. There were a total of 116 positions in the final dataset. Phylogenetic analyses were conducted in MEGA4 (Kumar *et al.*, 1994). (B) The SMART (Simple Modular Architecture Research Tool) prediction shows the presence of the BAH domain spanning between 44^th^ and 179^th^ amino acids at the N-terminal of CaORC1 and (C) The AAA+ domain in CaORC4 protein, the purple box represents the low complexity region (LCR). The LCR may be involved in flexible binding associated with specific functions but also that their positions within a sequence may be important in determining both their binding properties and their biological roles (Coletta *et al.*, 2010). (D) Comparative domain architecture of ORC proteins in *S. cerevisiae* and *C. albicans.* The red box denotes the BAH domain, the grey box is the AAA+ domain, cyan bar represents the AT-hook motif, black bar represents the Walker motifs, dark blue bar represents the PIP motif, yellow bar represents the MIR motif and the green bar represents the PEST motif. (E) Molecular Phylogenetic analysis of ORC proteins in the CTG clade by Maximum Likelihood method. The evolutionary history was inferred by using the Maximum Likelihood method based on the JTT matrix-based model (Tamura *et al.*, 2007). The tree with the highest log likelihood (-14518.99) is shown. Initial tree(s) for the heuristic search were obtained automatically by applying Neighbor-Join and BioNJ algorithms to a matrix of pairwise distances estimated using a JTT model, and then selecting the topology with superior log likelihood value. The tree is drawn to scale, with branch lengths measured in the number of substitutions per site. The analysis involved 29 amino acid sequences. All positions containing gaps and missing data were eliminated. There were a total of 237 positions in the final dataset. Evolutionary analyses were conducted in MEGA6 (Tamura *et al.*, 2013). (F) The time tree molecular phylogenetic analysis of ORC proteins in the CTG clade by the Maximum Likelihood method. The timetree shown was generated using the RealTime method (Tamura *et al.*, 2012). Divergence times for all branching points in the topology were calculated using the Maximum Likelihood method based on the JTT matrix-based model (Jones *et al.*, 1992). The estimated log likelihood value of the topology shown is -14518.99. The tree is drawn to scale, with branch lengths measured in the relative number of substitutions per site. The analysis involved 29 amino acid sequences. All positions containing gaps and missing data were eliminated. There were a total of 237 positions in the final dataset. Evolutionary analyses were conducted in MEGA6 (Tamura *et al.*, 2013). (G) 3D model of CaORC4 with DNA. (H) 3D model of CaORC4 with Walker A bound to ATP sphere, Walker B and R finger motifs.

## Description

DNA replication in eukaryotes is initiated by the orchestrated assembly and association of initiator proteins on the replication origins. The hunt for initiator proteins in higher eukaryotes picked up pace after the discovery of the Origin Recognition Complex (ORC) comprising of six protein subunits of the ORC1-6 complex in budding yeast (Bell and Stillman, 1992). In *Candida albicans*, a human fungal pathogen, the ORC (CaORC1-6) and their associated proteins were identified by a BLAST analysis using the *S. cerevisiae* proteins as the query sequences in the Candida Genome Database (CGD). The phylogenetic analysis suggests that in spite of limited amino acid sequence similarity with their counterparts in other organisms, the CaORC proteins share most of the functional domains with them. Interestingly, the amino acid sequences of CaORC1, 2 and 4 share higher degree of similarities than CaORC3, 5 and 6 to those of *S. cerevisiae*. CaORC1, 4 and 5 tend to be homologous to the mammalian counterparts. Although, in general, the CaORC proteins show limited sequence similarities with their counterparts in various species, CaORC1, 2 and 6 show maximum similarities to their *S. cerevisiae* counterparts while CaORC3, 4 and 5 appear to be more similar to those of mammals which is evident from the phylogenetic map (Figure 1A, Extended Data Tables 1-3).

The Expasy PROSITE tool predicts the presence of an evolutionarily conserved Bromo-Adjacent Homology (BAH) domain spanning the region between 44^th^ and 179^th^ amino acids at the N-terminal of CaORC1 (Figure 1B). The BAH domain is involved in protein-protein interactions and has been found to be important in DNA methylation, replication and transcriptional regulation (Callebaut *et al.*,1999). The BAH domain present in CaORC1 along with the highly conserved basic residues (K-362 and R-367) (Kawakami *et al.*, 2015) in its AAA domain is likely to play a key role in ORC-origin binding in *C. albicans*. The ATPase activity is indispensable for the origin-ORC association and henceforth for the establishment of the pre-initiation complex. Like metazoans, the CaORC subunits 1, 4, and 5 and CaCdc6 containing AAA+ domains are likely to be engaged in ORC assembly and consequent MCM recruitment. The presence of AAA+ domains, along with Walker A and B motifs provides a plausible explanation for the significance of these domains in CaORC4 in DNA replication (Extended Data Tables 4-6). From the conservative nature of the tyrosine residue between the Walker B motif and sensor I domain observed in humans and yeast, it is possible that the presence of this residue in CaORC4 (Tyr^273^) might play a regulatory role in the cell cycle (Extended data Figure 1). CaORC1 and CaORC4, each contains a consensus AAA+ domain (420-571 a.a. in CaORC1; 139-318 a.a. in CaORC4) (Figure 1B, 1C and 1D), which belongs to the AAA+ family that is pivotal to the initiation of eukaryotic DNA replication. There is an amino acid residue Tyr^174 ^in human ORC4 (Tyr^232^ in *S. cerevisiae*) that is found between the Walker B motif and sensor I of the AAA+ domain which may be responsible for interacting with a conserved arginine residue on an adjacent helix structure of ORC4 (Bell and Dutta, 2002, Wigley 2009, Duncker *et al.*, 2009, Kawakami and Katayama, 2010, Bell 2002, Guernsey *et al.*, 2011). Identification of missense mutation in ORC4, Y174C in humans is reported in Meier-Gorlin syndrome (Bicknell *et al.*, 2011, Guernsey *et al.*, 2011) whereas in yeast, ORC4 mutation, Y232C resulted in slower growth rate with G1 to S phase transition defects (Ladha 2011) and locus specific chromosome breakage (Sanchez *et al.*, 2017). This residue is present in CaORC4 (Tyr^273^) too probably doing a similar function. SNAP2 analysis suggests that this residue is crucial and whose mutation might cause functional defects in the protein (Extended data Figure1).

The Walker motifs are present in CaORC1, CaORC4 and CaORC5 (Walker B is absent in CaORC5) (Extended Data Table 4). Besides CaORC1, the perfect signature of the Walker motif is found in CaORC4 with a putative Walker A motif (147-153 a.a) and a putative Walker B motif (410-426 a.a), the amino acid sequences for which are shown in Extended Data Table 5. These motif signatures seem to be more closely related to the metazoan/higher eukaryotic sequences. A conserved Proliferating Cell Nuclear Antigen (PCNA) binding motif called the PCNA-interacting protein (PIP) box (QXXMXXFFFY) is found in the CaORC1 protein (524-536 a.a). Of the CaORC proteins, the PIP box is found to be unique to CaORC1. A conserved peptide motif named MIR (MOD1 interacting region – PXVHH) which is essential for their interaction with MOD1, a serotonin-gated chloride channel that modulates locomotory behavior in *C. elegans* (Ranganathan *et al.*, 2000) is found in CaORC3 protein (435-448 a.a). *In vivo* studies demonstrate that the MIR domain of ORC3 is important in the HP1α interaction (Prasanth *et al.*, 2010) suggesting a non-replicative role for CaORC3 too.

CaORC2 (130-172 a.a.) and CaORC3 (6-33 a.a.) contain PEST motif. Analysis of PEST signals in human and mouse ORC proteins suggests that only ORC1 is targeted for ubiquitination which is likely to hold good for all mammals (Li and DePamphilis, 2002). The domains of CaORC proteins are compared with other eukaryotes and are tabulated in Extended Data Table 6 and compared with *S. cerevisiae* in Figure 1D. The evolution of phospho regulation pattern in replication proteins of various yeast species including *C. albicans* is reported (Beltrao *et al.*, 2009).

Experimental evidence would be required to find out if some or all of these subunits are involved in ATP binding and hydrolysis in *C. albicans*. The cryo-EM structural studies demonstrate that Cdt1 helps in the recruitment of Cdc6 and Mcm2-7 thus forming the ORC-Cdc6-Cdt1-Mcm2-7 (OCCM) intermediate (Yuan *et al.*, 2017). The absence of a Cdt1 homolog in *C. albicans* suggests that this important task may be accomplished by a different mechanism/factor (Extended Data Tables 6 and 7). The unique presence of the PEST motif in CaORC2 and CaORC3 indicates that these components might be facilitating ORC turnover.

Moreover, the CaORC proteins are also compared across CTG clade and other yeast species to provide a robust roadmap for further comparative yeast subphylum analysis (Figure 1E and F and Extended data Figure 2). The ORC1, ORC2 and ORC5 proteins from yeast to humans are found to have common nodes. Subsequently, the ORC proteins from the related species of *C. albicans* in the CTG clade were also compared and a phylogenetic tree was constructed (Figure 1E). The time tree demonstrates the diversification rate of these ORC proteins across the species of which ORC1 and ORC4 seem to be older than their counterparts (Figure 1F). In order to understand the sequence identity of the ORC sequences across various yeast species, Sequenceserver (http://blast.wei.wisc.edu/) (Priyam *et al.*, 2019, Shen *et al.*,2016) was used across 86 publicly available yeast genomes (Extended data Figure 2; Extended Dataset).

We used I-TASSER (Iterative Threading ASSEmbly Refinement) (Zhang 2008) for structure prediction of CaORC proteins. Of all the CaORC proteins, CaORC4 was found to be one of the putative candidates for further fine refinement studies of the protein structure due to its higher Cscore (combined measure, See Methods section) which indicates a better confidence in predicting the function using the template (Extended Data Table 8). Hence, we proceeded for predicting the structure of CaORC4 using Phyre. We were able to build the 3D protein structure of CaORC4 only whereas the other CaORC proteins did not have good homology with the known PDB (Protein Data Bank) structures (Figure 1G and 1H). From our *in silico* analysis of interactive studies, it is evident that CaORC3, CaORC5 and CaORC6 do not interact with the other ORC counterparts. It is possible that only CaORC1, CaORC2 and CaORC4 would be involved in DNA binding during the process of DNA replication and the other counterparts may aid in tethering or in conformational organization. It is not known as to whether the CaORC exists as a complex which requires experimental validation. CaCdc6 and CaMCM4, the apparent common binding partners of CaORC1, CaORC2 and CaORC4 and many MCMs are also predicted to play important role(s) in preRC assembly and functioning. We also find a potential ATP binding site in CaORC4 which might help in the regulation of origin binding. The mode of ORC assembly at origins in *C. albicans* might be different from that in other yeasts. The *in silico* detection of the presence of AAA+ ATPase and Walker motifs in CaORC4 and its likely interaction with MCM proteins suggest that CaORC4 might be involved in stable binding to origin DNA and loading MCM proteins to origins. While possibilities of a physical association between CaORC4 and other CaORC proteins were not obvious (Extended Data Table 9), the role of some unknown factors mediating ORC assembly in *C. albicans* is not ruled out. CDC6, MCM4 and MCM proteins interact with CaORC1, CaORC2 and CaORC4. In absence of a direct interaction of CaORC4 with other ORC counterparts, these proteins might be mediating interaction between them. Moreover, the absence of Cdt1 in *C. albicans* might provide an additional role for CaORC4. The recent cryo-EM data of the ORC in yeast (Yuan *et al.*, 2017) and humans (Li *et al.*, 2018) might open up more avenues in understanding the structural dynamics of this complex in other species too.

## Methods

**Annotation of *C. albicans* pre-RC genes**

The genome of *C. albicans* (http://www.candidagenome.org/) was searched for homologs of pre-RC complex genes using BLAST (Altschul *et al.*, 1997). Alignment of pre-RC gene sequences from Candida and its homologs in other eukaryotic organisms was carried out using the ClustalW algorithm (Thompson *et al.*, 1994). The pairwise percent identity scores are calculated by the number of identities between the two sequences, divided by the alignment length in terms of percentage.

**Phylogenetic analysis**

Phylogenetic analysis was performed with the MEGA4 program (Kumar *et al.*, 1994, Tamura *et al.*, 2007). ThePhylogenetic tree of ORC proteins was drawn to scale, with branch lengths in the same units as those of the evolutionary distances used to infer the phylogenetic tree. The evolutionary distances were computed using the Poisson correction method (Zuckerkandl and Pauling, 1965) and are in the units of the number of amino acid substitutions per site. All positions containing gaps and missing data were eliminated from the dataset (Complete deletion option). There were a total of 116 positions in the final dataset. Phylogenetic analyses were conducted in MEGA4. The molecular phylogenetic analysis of ORC proteins in the CTG clade was performed by Maximum Likelihood method. The evolutionary history was inferred by using the Maximum Likelihood method based on the JTT matrix-based model (Tamura *et al.*, 2007). The tree with the highest log likelihood (-14518.99) is shown. Initial tree(s) for the heuristic search were obtained automatically by applying Neighbor-Join and BioNJ algorithms to a matrix of pairwise distances estimated using a JTT model, and then selecting the topology with superior log likelihood value. The tree is drawn to scale, with branch lengths measured in the number of substitutions per site. The analysis involved 29 amino acid sequences. All positions containing gaps and missing data were eliminated. There were a total of 237 positions in the final dataset. Evolutionary analyses were conducted in MEGA6 (Tamura *et al.*, 2013). The timetree of the ORC proteins in CTG clade was generated using the RealTime method (Tamura *et al.*, 2012). Divergence times for all branching points in the topology were calculated using the Maximum Likelihood method based on the JTT matrix-based model (Jones *et al.*, 1992). The estimated log likelihood value of the topology shown is -14518.99. The tree is drawn to scale, with branch lengths measured in the relative number of substitutions per site. The analysis involved 29 amino acid sequences. All positions containing gaps and missing data were eliminated. There were a total of 237 positions in the final dataset. Evolutionary analyses were conducted in MEGA6 (Tamura *et al.*, 2013).

***In silico* analysis**

The putative protein sequences whose theoretical characteristics were obtained using several programs in the ExPASy (Expert Protein Analysis System) server of the Swiss Institute of Bioinformatics (www.expasy.ch/tools/). Protein sequences were entered into MotifScan (pattern searches), ProDOM (protein domain identification), Interpro (protein domain and pattern search identification), NetPhos (prediction sites for phosphorylation) and PESTfind (identification of PEST sequences), SMART (prediction of protein domain architecture) and Phyre (secondary structure prediction) for the analyses. To determine the sequence identity of CaORC across 86 diverse publicly available yeast databases, a TBLASTN was performed in the Sequenceserver (http://blast.wei.wisc.edu/) with CaORC proteins as the query sequence by clicking the option (_all_merged_public.fas) in the nucleotide databases (Priyam *et al.*, 2019, Shen *et al.*,2016) and the percent identity was plotted against the species using Graphpad Prism (Swift M.L., 1997).

**Phyre structure prediction parameters**

Cscore^GO ^is a combined measure for evaluating global and local similarity between query and template protein. This score ranges from 0-1 where a higher value indicates a better confidence in predicting the function using the template. Cscore^LB ^is the confidence score of predicted binding site of the protein with values ranging between 0-1. Higher the score more reliable is the ligand binding prediction.

**Variant analysis using SNAP2**

SNAP2, an open source online prediction platform uses neural network to distinguish between effect and neutral variants/non-synonymous SNPs by taking a variety of sequence and variant features into account (Hecht *et al.*, 2015). The CaORC protein sequences were loaded on to the SNAP2 server and the results were obtained in the form of a heatmap and the score of the variants were listed in the form of a table. The value -100 denotes neutral and +100 denotes effect.
